# PRKCB is relevant to prognosis of lung adenocarcinoma through methylation and immune infiltration

**DOI:** 10.1111/1759-7714.14466

**Published:** 2022-05-13

**Authors:** Jinjie Wang, Muqi Shi, Haijian Zhang, Hao Zhou, Zhanghao Huang, Youlang Zhou, Jiahai Shi

**Affiliations:** ^1^ Nantong Key Laboratory of Translational Medicine in Cardiothoracic Diseases and Research Institution of Translational Medicine in Cardiothoracic Diseases Affiliated Hospital of Nantong University Nantong China; ^2^ Department of Thoracic Surgery Affiliated Hospital of Nantong University Nantong China; ^3^ Medical College of Nantong University Nantong China; ^4^ Research Center of Clinical Medicine Affiliated Hospital of Nantong University Nantong China; ^5^ School of Public Health Nantong University Nantong China

**Keywords:** bioinformatics analysis, DNA methylation, immune infiltration, LUAD, survival analysis

## Abstract

**Background:**

Lung adenocarcinoma (LUAD) is one of the tumor‐related diseases with high morbidity worldwide. Epigenetic modifications such as DNA methylation changes may involve in tumorigenesis. This study aimed to explore new biomarkers that have prognostic significance of LUAD.

**Methods:**

First, we downloaded the gene expression and methylation data set from Gene Expression Omnibus. R software was then used to identify abnormally methylated differentially expressed genes (MDEGs). Next, R package Cluster Profiler was used to analyze the enrichment and pathway of the MDEGs. Analysis using STRING revealed the protein–protein interaction network. The result was then visualized by Cytoscape and obtained 10 hub genes. Afterward, they were further verified by The Cancer Genome Atlas to select candidate genes. Moreover, quantitative real‐time polymerase chain reaction (qRT‐PCR) and immunohistochemistry were used to verify the expression and prognostic value of candidate genes in LUAD patients.

**Results:**

The results showed that the expressions of ADCY5 and PRKCB are indeed related to LUAD. The clinical relevance to PRKCB was confirmed by its clinical correlation analysis. Gene set enrichment analysis (GSEA) and tumor immune estimation resource (TIMER) tumor immune correlations showed that PRKCB is involved in the cancer‐related Kyoto Encyclopedia of Genes and Genomes pathway and is involved in immune infiltration. It was also verified by qRT‐PCR and immunohistochemistry that PRKCB was lowly expressed in LUAD patients and correlated with prognosis.

**Conclusions:**

PRKCB is relevant to prognosis of LUAD through methylation and immune infiltration.

## Graphical abstract

This study identified 124 abnormally methylated differentially expressed genes (MDEGs) from GEO dataset. A protein–protein interaction (PPI) network was established to screen out 10 hub genes. The gene expression and methylation levels were further verified by TCGA to obtain the target gene PRKCB. TIMER analysis indicated that PRKCB was involved in the immune infiltration of LUAD. qRT‐PCR and IHC indicated that PRKCB was underexpressed in LUAD and correlated with prognosis.

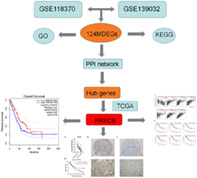

## INTRODUCTION

Lung cancer can be divided into small‐cell lung cancer (SCLC, about 15%) and non–small‐cell lung cancer (NSCLC, ~85%) according to histopathology. Lung adenocarcinoma (LUAD) is one of the histological subtypes of NSCLC, and its incidence has increased significantly in recent years.^1^ LUAD is usually diagnosed in advanced stages. However, if diagnosed early, survival rate of LUAD patients can be greatly extended. Therefore, to reduce the mortality of LUAD, effective early identification methods and related biomarkers are urgently needed. Currently, in addition to low‐dose tomography widely used in lung cancer screening and postoperative monitoring, potential biomarkers such as autoantibodies, complement fragments, microRNAs, circulating tumor DNA, DNA methylation, and blood protein profiles have attracted widespread attention.^3^

DNA methylation is a form of epigenetic modification that can change genetic performance without changing the DNA sequence. This is one of the current research hotspots in tumor and molecular biology.^4^ Recent studies have shown that changes in the methylation pattern of tumor cells can be divided into two types: hypomethylation of oncogenes leads to activation of oncogenes, and increased levels of DNA methylation in specific regions cause inactivation of tumor suppressor genes.^5^ Because DNA methylation often occurs in lung cancer,^6^ we sought to discover new DNA methylation biomarkers in LUAD patients, which may become a prognostic factor for LUAD patients.

With the advantage of big data networks, convenient and public databases such as Gene Expression Omnibus (GEO)^7^ and The Cancer Genome Atlas (TCGA),^8^ which contain gene expression levels and methylation characteristics of various tumors and normal samples, makes it possible to select the most detectable organism from a large number of potential markers.

In this study, we sought to identify genes that are abnormally methylated in LUAD through systematic bioinformatics analysis, which is a more accurate analysis of huge biological and genomic data. To increase the persuasive power of this study, we verified by quantitative real‐time polymerase chain reaction (qRT‐PCR) that PRKCB was indeed downregulated in LUAD tissues. At the same time, we further verified by immunohistochemistry (IHC) that LUAD patients with low PRKCB expression level had worse overall survival (OS). In short, PRKCB may act on LUAD patients through methylation and immune infiltration.

## MATERIALS AND METHODS

### Data source

Two datasets associated with LUAD were downloaded from GEO (https://www.ncbi.nlm.nih.gov/geo/): GSE118370 (including six LUAD tissues and six paired normal lung tissues) and methylation dataset GSE139032 (including 77 LUAD samples and 77 matched non‐malignant lung samples).

### Identification of methylated differentially expressed genes (MDEGs) and functional analysis

The raw data of GSE118370 were preprocessed and normalized by using the affy package under the R environment (https://www.r-project.org/). After pretreatment, we used the limma package to identify genes differentially expressed in LUAD tissues with |logFC| >1 and adjusted *p* value <0.05. Meanwhile, data of GSE118370, which related to the methylation expression level of genes, were first standardized and normalized in the R environment using the wateRmelon package. Next, we took β value >0.2 and adjusted *p* value <0.05 as the standard of abnormal methylation. Finally, we used the online Wayne diagram to cross‐contrast the DEGs with abnormally methylated to obtain MDEGs. The Gene Ontology (GO) and Kyoto Encyclopedia of Genes and Genomes (KEGG) pathways of MDEGs were performed by R package cluster profiler based on org.Hs.eg.db database.^9^, ^10^, ^11^, ^12^ The GO and KEGG analysis results were visualized using the enrichplot and the GOplot package.^13^ GO analysis includes biological processes (BP), molecular functions (MF), and cellular components (CC).

### Construction of protein–protein interaction (PPI) network

Potential relationships between MDEGs were identified by the online STRING (https://www.string-db.org/), which is a database that uses bioinformatics to predict the PPI network.^14^ Next, Cytoscape (https://cytoscape.org) was used to visualize the PPI and further find hub genes.^15^ The top 10 hub genes were then identified using the cytoHubba plugin and the Maximal Clique Centrality method. In addition, core modules of the PPI network with degree cutoff = 2, node score cutoff = 0.2, and *K*‐core = 2 were selected by the plug‐in Molecular Complex Detection (MCODE) in Cytoscape software.

### Expression and methylation levels of hub genes in TCGA

The Gene Expression Profiling Interactive Analysis (GEPIA) (http://gepia.cancer-pku.cn) combined with TCGA database was used to further confirm the expression levels of hub genes between LUAD and normal tissues.^16^ The online The University of Alabama at Birmingham Cancer data analysis portal (UALCAN) (http://ualcan.path.uab.edu) was also used to confirm the methylation levels of hub Genes between LUAD and normal tissues combined with the TCGA database.^17^ In addition, cBioPortal for Cancer Genomics (https://www.cbioportal.org) was used to further analyze the correlation between expression and methylation of hub genes.

### Survival and prognosis analysis

GEPIA was used to evaluate the relationship between the expression of candidate genes in LUAD and survival rate. With the help of the TCGA database, OS of LUAD patients can be assessed based on the different expression levels of each gene.

### Analysis of clinical relevance of candidate genes in TCGA

All gene expression data (594 cases) and corresponding clinical information of LUAD can be downloaded from the TCGA official website. Samples with incomplete clinical information were exclued when investigating clinical relevance.

### Gene set enrichment analysis of PRKCB

Genes was classified by gene set enrichment analysis (GSEA) according to the degree of differential expression in two types of samples, and then checks whether the top or end of the preset list is enriched with the preset gene set.^18^ GSEA first, generated a ranked table of all genes according to the correlation between the expression of all genes and PRKCB, and second, divides the expression of PRKCB into high expression group (PRKCB‐h) and low expression group (PRKCB‐l) according to the median. At last, GSEA was then performed to clarify the significant survival differences between PRKCB‐h and PRKCB‐1. Each analysis was performed 1000 times. The nominal *p* value and normalized enrichment score (NES) were used to rank each phenotype enrichment pathway.

### Correlation analysis of PRKCB and immune cell infiltration

Tumor immune evaluation resources (TIMER) (https://cistrome.shinyapps.io/timer) is a free online website based on the TCGA database that uses statistical methods to detect the infiltration of immune cells in tumor tissues and its impact on the prognosis of patients.^19^ The immune cells in this study included CD4^+^ T cells, CD8^+^ T cells, B cells, macrophages, neutrophils, and dendritic cells.

### Clinical samples collection

We collected 20 tumor tissues (tumor) and adjacent normal tissues (normal) from LUAD patients, which were taken from the Affiliated Hospital of Nantong University and stored at −80°C for subsequent RNA extraction. In addition, we retrospectively studied a tissue microarray (TMA) of 60 tissues of LUAD patients who underwent surgical treatment at the Affiliated Hospital of Nantong University from January 2010 to June 2017. We extracted the clinical characteristics of the LUAD patients from the medical record, including age, gender, smoking history, differentiation, and pathological TNM stage. This experimental protocol was approved by the Ethics Committee of the Affiliated Hospital of Nantong University.

### RNA extraction and qRT‐PCR

We selected 20 pairs of LUAD tumor tissues and paired normal lung tissues clinically, extracted total RNA from them with TRIzol reagent (Life Technologies) and transcribed to complementary DNA (cDNA) using the PrimeScript RT reagent kit (Takara). Finally, qRT‐PCR was used to analyze the expression levels of candidate genes in tissues. We set the reaction conditions as follows: incubate at 95°C for 2 minutes and then perform 45 cycles at 95°C for 5 seconds and 60°C for 30 seconds. The analysis software (Eppendorf) displayed the cycle threshold of each reaction. The GAPDH gene served as an internal control. The primers of PRKCB were as follows: forward 5′‐CGTCCTCATTGTCCTCGTAA‐3′ and reverse 5′‐TGTCTCATTCCACTCAGGGTT‐3′.

### IHC

IHC method was used to detect the expression level of PRKCB in paraffin‐embedded LUAD specimens. The samples were first incubated with rabbit anti‐PRKCB antibody (1:200), and then incubated with goat anti‐rabbit secondary antibody (1:500) for secondary staining. Finally, a microscope (Leica DMR 3000; Leica Microsystem) was used to capture images of each slice at a magnification of 200‐fold. PRKCB (brown)‐positive staining is mainly located in the cytoplasm. It was scored according to staining intensity and percentage of PRKCB‐positive tumor cells. The median was used as the cutoff value for high or low PRKCB expression.

### Statistical analysis

The Wilcoxon signed‐rank test and logistic regression were used to analyze the relationship between clinicopathological characteristics and candidate genes. Cox regression was used to assess the clinicopathological features associated with overall survival in TCGA patients. Multivariate Cox analysis was used to compare the effect of candidate gene expression on survival and other clinical characteristics. The hazard ratio referred to the risk of death in LUAD patients as the value of each risk factor increased. Statistical significance of qRT‐PCR was determined using Student's *t*‐test. Relationships between PRKCB expression and clinicopathological characteristics were evaluated using the χ2 test or Fisher's exact test. Kaplan–Meier method was used to construct OS curve, and log‐rank test was used to analyze the difference between the curves.

## RESULTS

### Identification of MDEGs

**FIGURE 1:**
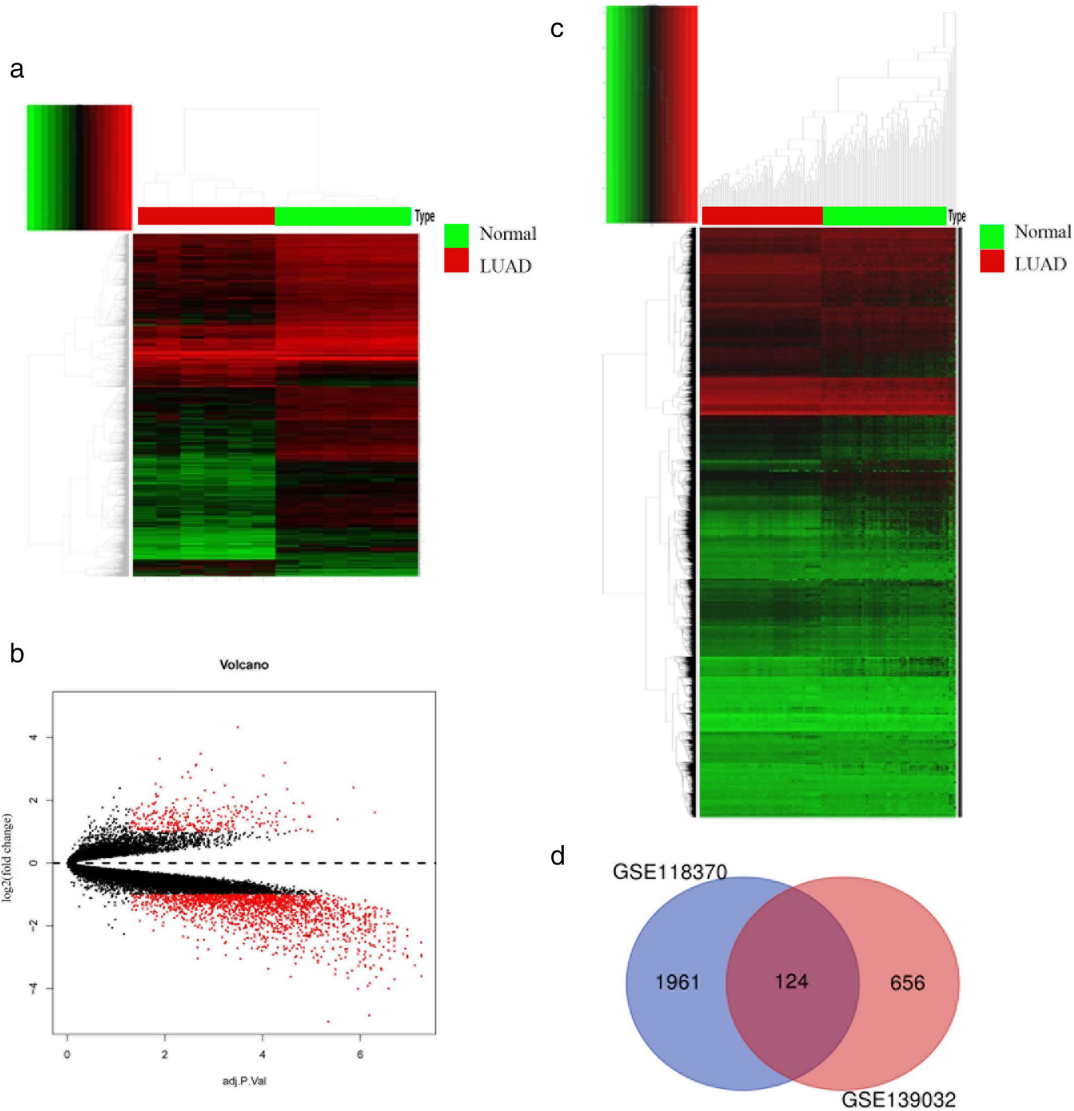
Identification of abnormally methylated differentially expressed genes (MDEGs) in lung adenocarcinoma (LUAD): (a) heat map of DEGs in GSE118370. Red, upregulated genes in LUAD; green, downregulated genes in LUAD. (b) Volcano plot of DEGs in GSE118370. (c) Heat map of DEGs in GSE139032. Red, hypermethylated genes in LUAD; green, hypomethylated genes in LUAD. (d) Venn diagram of MDEGs.

To find genes with differential expression of LUAD, we first downloaded GSE118370, which contains all gene expression datasets from LUAD tissues and paired normal lung tissues, from GEO. We identified 2085 significant DEGs (301 upregulations, 1784 downregulations) (Figure 1(a),(b)) in LUAD from GSE118370. At the same time, the GSE139032 data was processed for the methylation data of LUAD to further obtain 780 methylation differential genes (Figure 1(c)) in LUAD. Next, the genes screened from the two gene sets described above were jointly imported into a Venn diagram (Figure 1(d)). This resulted in 124 overlapping MDEGs.

### GO and KEGG analysis of MDEGs

**FIGURE 2:**
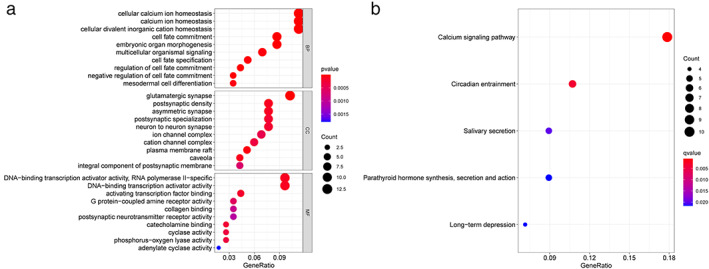
Gene Ontology (GO) and Kyoto Encyclopedia of Genes and Genomes (KEGG) pathway analyses results of the methylated differentially expressed genes with a fold change of more than 2. BP, biological process; CC, cellular component; MF, molecular function. Log10 fold‐changes are used as parameters.  (a) The top 10 significant GO terms. (b) The top 20 significant KEGG terms.

Next, we sought to find out the common biological function of these 124 MDEGs. Therefore, we used R package Cluster Profiler for GO and KEGG analysis. As we can see from Figure 2(a), the five most important functions of BP were cellular calcium ion homeostasis, calcium ion homeostasis, cellular divalent inorganic cation homeostasis, embryonic organ development, and cell fate commitment. The top five functions of CC were glutamatergic synapse, postsynaptic density, asymmetric synapse, postsynaptic specialization, and neuron to neuron synapse. The top five positions of MF were DNA‐binding transcription activator activity, RNA polymerase II‐specific, DNA‐binding transcription activator activity, transcription coactivator activity, gated channel activity, and DNA‐binding transcription factor binding. In addition, KEGG pathway analysis showed that 124 MDEGs were concentrated in the calcium signaling pathway, circadian entrainment, salivary secretion, parathyroid hormone synthesis, secretion and action, and long‐term depression (Figure 2(b)).

### Construction of PPI network

**FIGURE 3:**
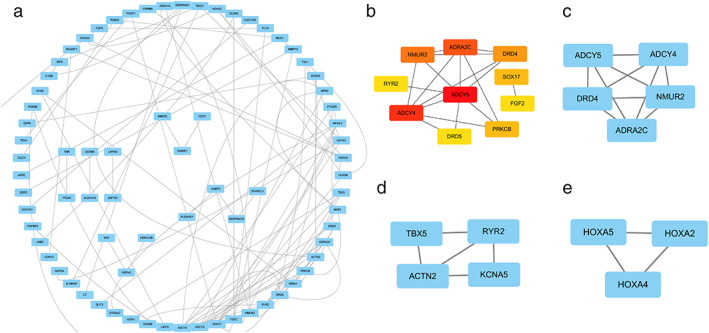
Protein–protein interaction (PPI) network and hub genes. Circles represented genes, and lines represented protein–protein associations of genes: (a) PPI network of methylated differentially expressed genes. (b) Top 10 hub gene network maps. (c) Module 1. (d) Module 2. (e) Module 3.

To find out key genes, we used an online STRING platform to identify potential protein interactions between these MDEGs. The resulting PPI network graph contains 123 nodes and 97 edges. We used Cytoscape software to visualize the PPI network (Figure 3(a)). Through the Cytoscape plugin, we identified the first 10 hub genes: RYR2, ADCY4, ADCY5, DRD5, PRKCB, NMUR2, ADRA2C, DRD4, SOX17, and FGF2. Meanwhile, the entire network was then analyzed using Cytoscape's MCODE plugin, which identified three subnetworks (Figure 3(b)–(e)).

### Expression and prognostic value in TCGA database

**FIGURE 4:**
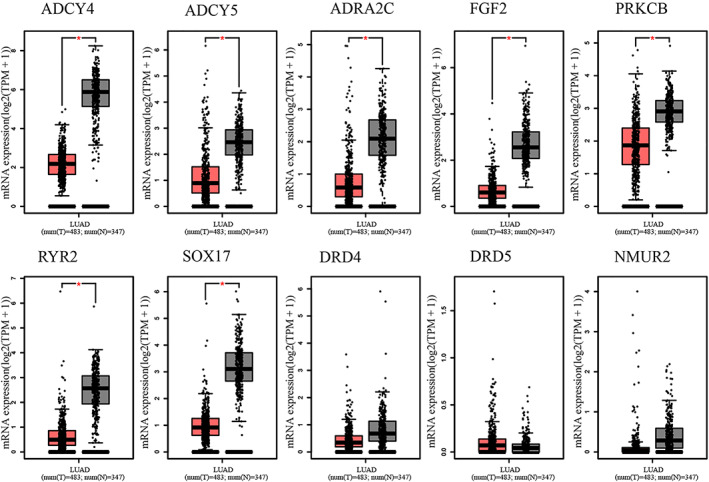
The expression of 10 hub genes through GAPIA database (left column reflects tumor data and right column reflects normal data, *p < 0.05)

**FIGURE 5:**
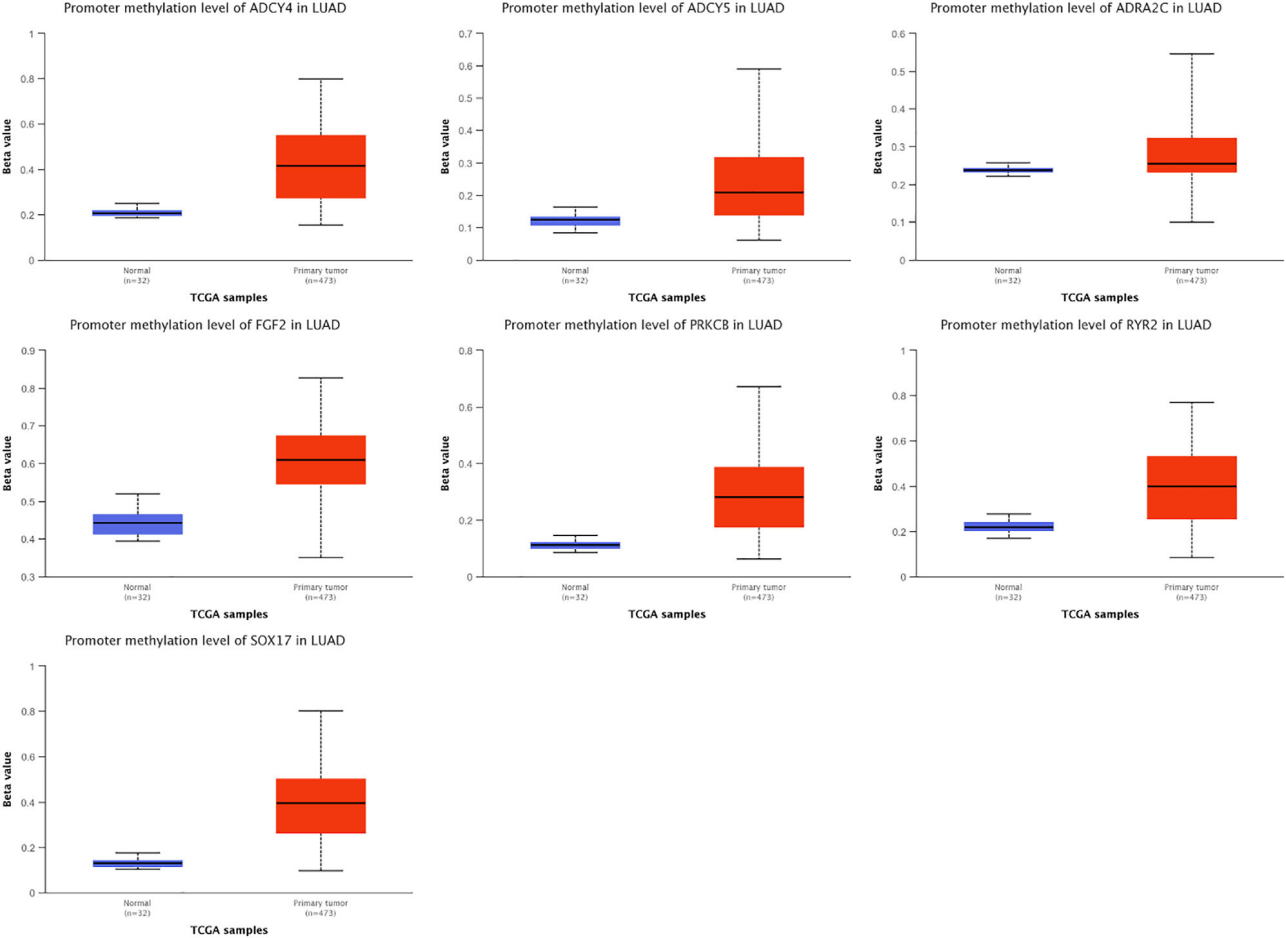
The methylation levels of seven hub genes through UALCAN database (left column reflects normal data and right column reflects tumor data, *p < 0.05)

**FIGURE 6:**
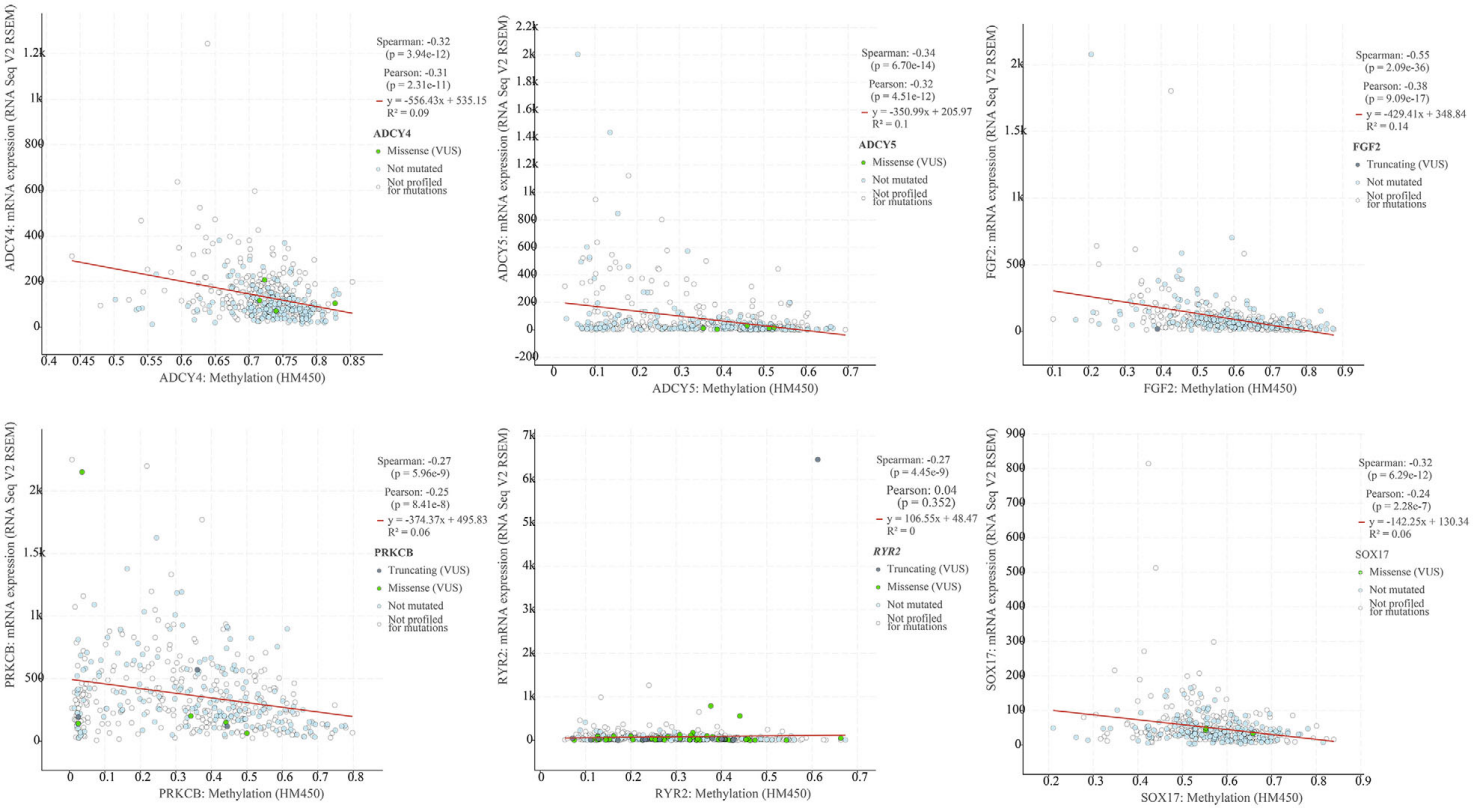
The relationship between the methylation level and expression of candidate genes was identified through the cBioPortal database. Spearman's correlation analysis was performed between methylation (horizontal axis) and mRNA expression (vertical axis). Spearman's correlation coefficient and *p* values are shown in the figure

**FIGURE 7:**
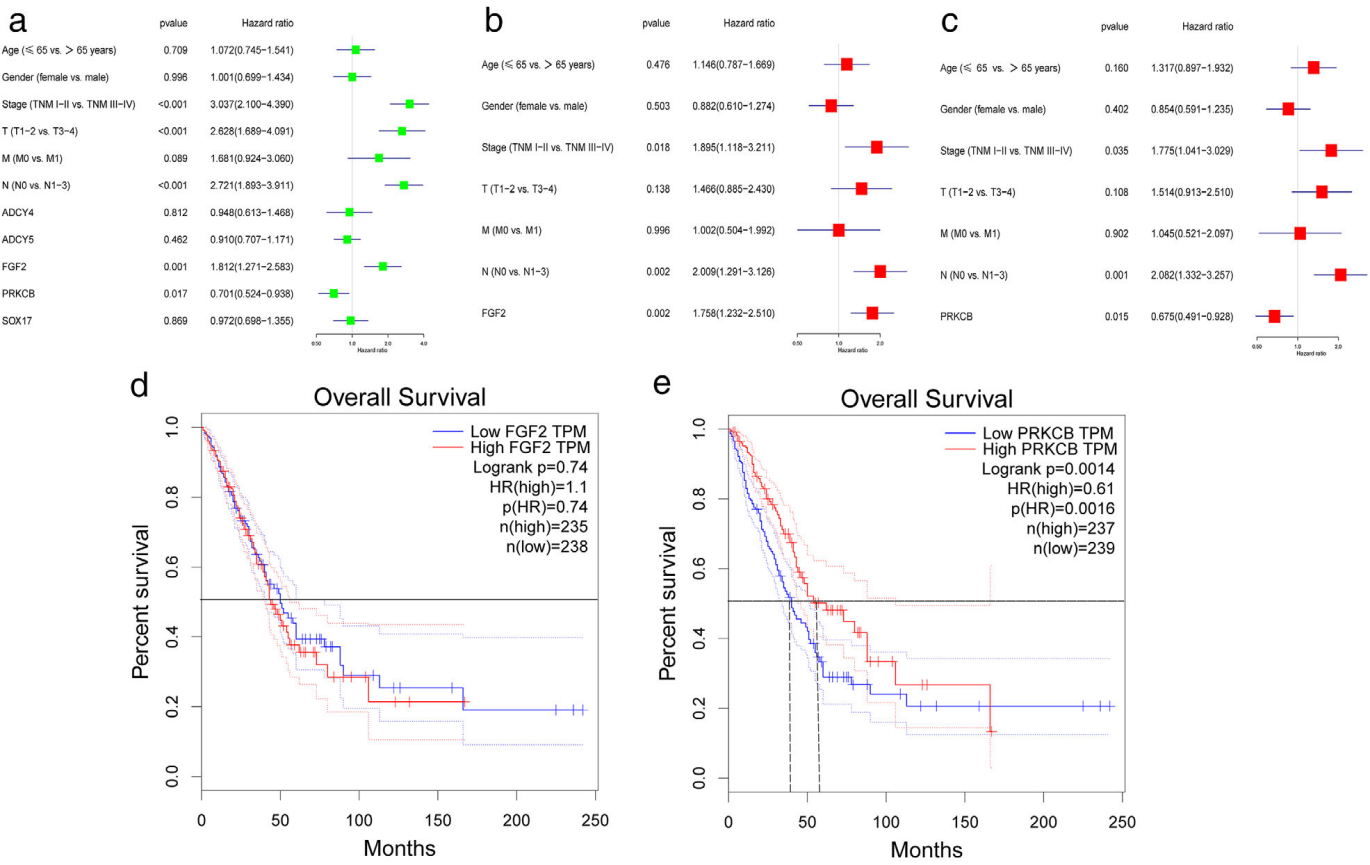
The correlation between the expression of candidate genes and clinicopathological features and prognosis of lung adenocarcinoma (LUAD). (a) Univariate cox analysis of overall survival of LUAD patients. (b),(c) Multivariate cox analysis showed the hazard ratio (HR) of different factors. (d),(e) The overall survival analysis of FGF2 and PRKCB were analyzed through the GEPIA database: based on the different expression levels of each gene, LUAD patients were divided into high and low groups to assess its prognostic value. (*p < 0.05)

**FIGURE 8:**
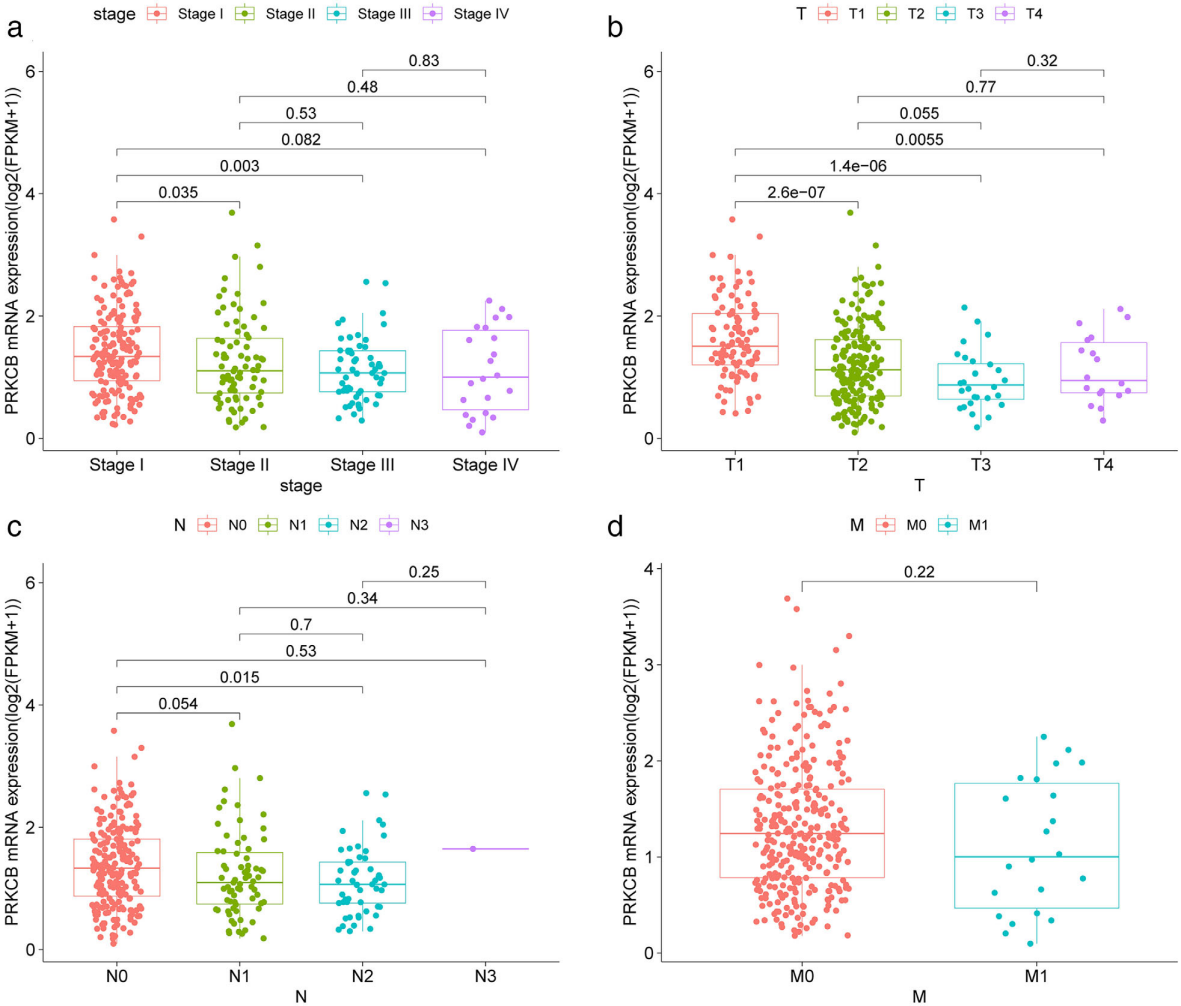
The relationship between PRKCB expression and clinicopathological characteristics. The clinicopathological features of lung adenocarcinoma include: (a) TNM stage, (b) *T* stage, (c) *N* stage, (d) *M* stage

To further verify the expression levels of candidate genes, we performed differential expression analysis using GEPIA online tool. It can be seen from the boxmap in Figure 4 that the expression of ADCY4 (fold change = 0.36), ADCY5 (fold change = 0.36), ADRA2C (fold change = 0.28), FGF2 (fold change = 0.24), PRKCB (fold change = 0.62), RYR2 (fold change = 0.19), and SOX17 (fold change = 0.30) in LAUD was lower than that in normal lung tissues. That is, these seven candidate genes may be suppressors in LUAD oncogene. There was no statistical difference in the expression level of the remaining three genes in the tumors and normal tissues. Hypermethylation in the DNA promoter region is an important regulatory mechanism for tumorigenesis, which is widely present in a variety of tumor suppressor genes. Therefore, we speculated that the downregulation of the expression of these seven genes in LUAD may be related to DNA promoter hypermethylation. We used the UALCAN to detect the methylation levels of these seven candidate genes in LUAD. The results showed that the methylation levels of ADCY4 (fold change = 2.02, *p* < 1E–12), ADCY5 (fold change = 1.85, *p* < 1E–12), FGF2 (fold change = 2.20, *p* = 1.62E–12), PRKCB (fold change = 2.55, *p* < 1E–12), RYR2 (fold change = 1.78, *p* < 1E–12), and SOX17 (fold change = 3.07, *p* < 1E–12) promoter regions in LUAD tissues were significantly higher than those in normal tissues (Figure 5). The methylation levels of ADCY4, ADCY5, FGF2, PRKCB, and SOX17 were negatively correlated with transcriptional expression (Figure 6). Next, we used the TCGA database to study the relationship between candidate gene expression levels and clinical characteristics. We performed univariate analysis of the clinicopathological characteristics and the five candidate genes to further screen prognostic genes (Figure 7(a)). Meanwhile, multivariate Cox analysis indicated that FGF2 and PRKCB were independent prognostic factors (Figure 7(b),(c)). Figure 7€ showed that the median survival time of patients with low expression of PRKCB was about 40 months, whereas the median survival time of patients with high expression of PRKCB was about 55 months. Therefore, we can conclude that highly expressed PRKCB in LUAD patients had better survival results (log rank *p* = 0.0014). Meanwhile, highly expressed FGF2 did not show the survival advantage (log rank *p* = 0.74) (Figure 7(d)). At the same time, we can see from Figure 8(a) that the expression of PRKCB in stage I was higher than that in stage II and stage III. The expression of PRKCB in *T*1 was significantly higher than that in *T*2 and *T*3 (Figure 8(b)). The expression of PRKCB in *N*2 was lower than that in *N*0 (Figure 8(c)). Although the median expression of PRKCB in *M*1 was lower than that in *M*0, it had no statistical significance (*p* > 0.05, Figure 8(d)). Therefore, we can preliminarily conclude that the expression of PRKCB was lower in the advanced stage than in the early stage.

### Identification of PRKCB‐Related signal paths with GSEA

**FIGURE 9:**
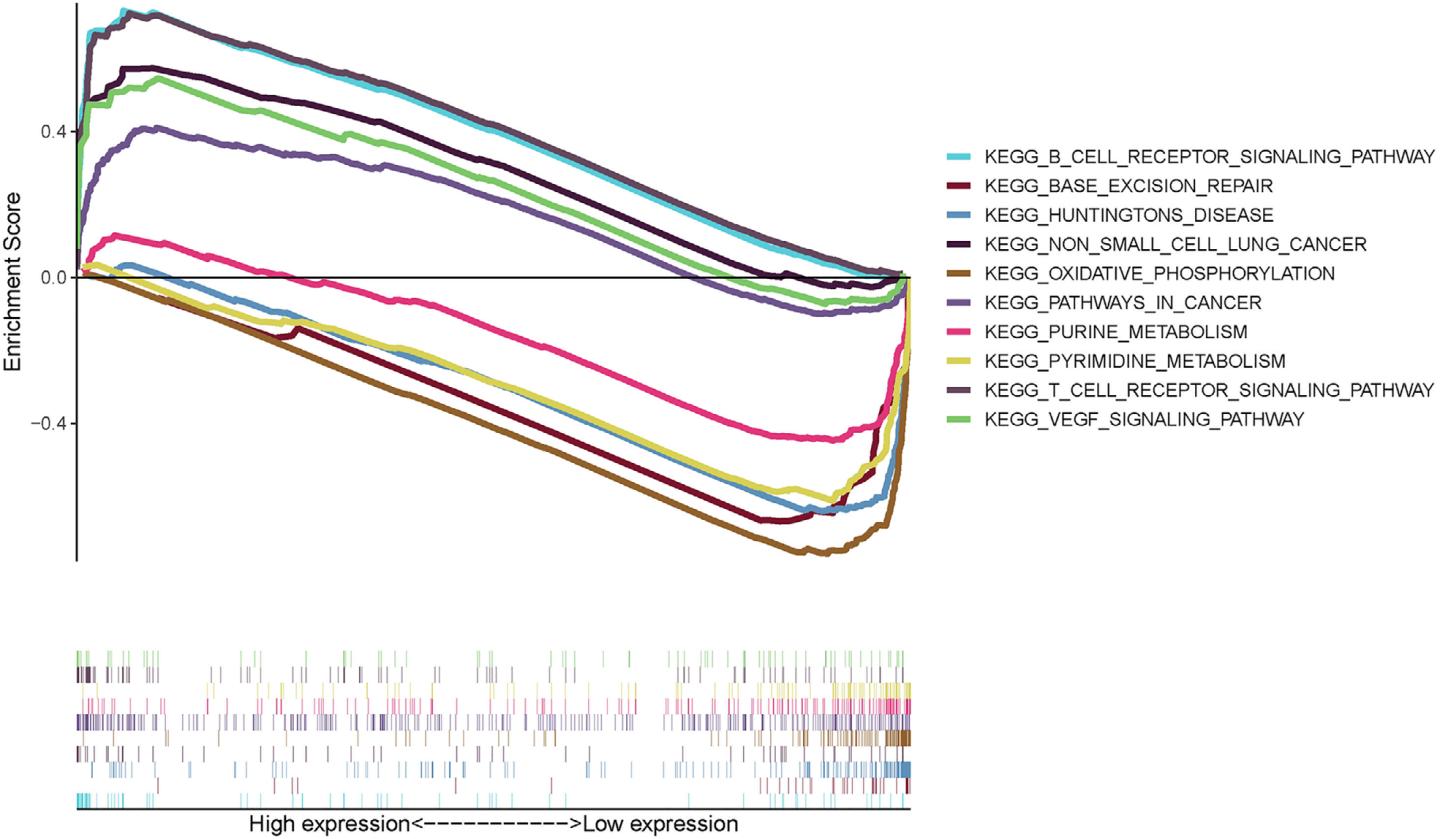
The PRKCB‐related lung adenocarcinoma gene set enrichment analysis (GSEA) was identified by TIMER software. The nominal *p*‐value and normalized enrichment score (NES) were used to rank each phenotypic enrichment pathway

To explore the potential mechanism of PRKCB in LUAD, KEGG was analyzed by GSEA method. As shown in Figure 9, genes related to high expression of PRKCB were concentrated on NSCLC, pathways in cancer, B cell receptor signaling, T cell receptor signaling, VEGF signaling pathway, and so on. In contrast, the PRKCB low expression related gene sets were rich in Huntington's disease, oxidative phosphorylation, purine metabolism, pyrimidine metabolism, and base excision repair. Taken together, these results suggested that PRKCB was indeed involved in the cancer‐related KEGG pathway.

### Correlation between PRKCB and tumor infiltrating immune cells

**FIGURE 10:**
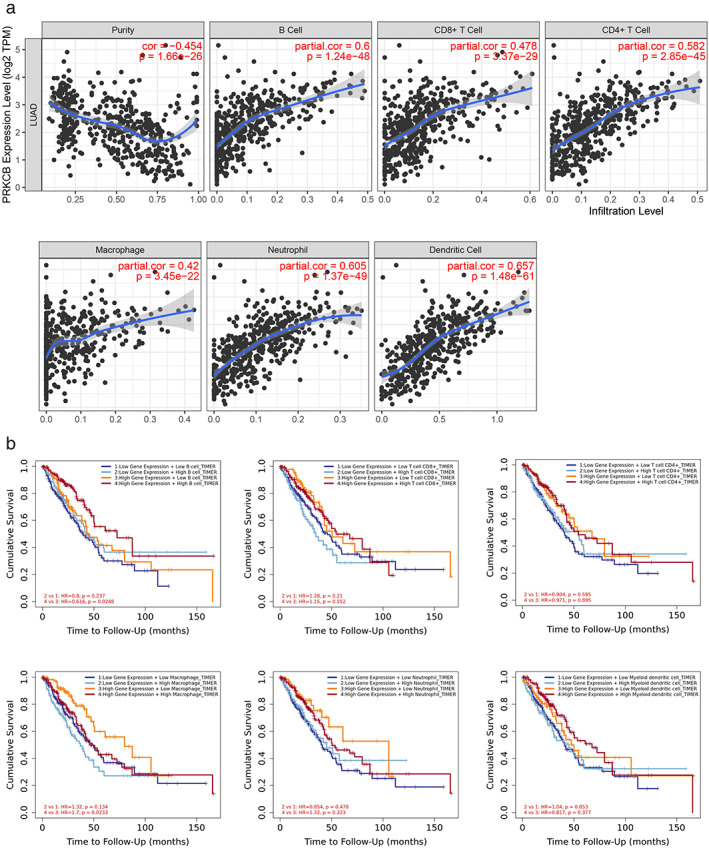
(a) Correlation between PRKCB expression and lung adenocarcinoma (LUAD) immune cell infiltration in TCGA database. (b) The effects of PRKCB and immune cells on the prognosis of LUAD patients were analyzed by the TIMER online database

It can be seen from GSEA that PRKCB expression was related to immune cell receptor signaling, and we used TIMER software to analyze the relationship between PRKCB expression and tumor infiltrating immune cells (Figure 10(a)). PRKCB expression and CD4^+^ T cells (cor = 0.582), CD8^+^ T cells (cor = 0.478), B cells (cor = 0.6), macrophages (cor = 0.42), neutrophils (cor = 0.605), and dendritic cells (cor = 0.657) had significant correlations. Among them, the expression of PRKCB is more correlated with neutrophils and dendritic cells. In addition, the expression of PRKCB was combined with the expression of each immune cell to analyze its influence on the prognosis of LUAD patients. We found that LUAD patients with high expression of PRKCB combined with high expression of B cells had better prognosis. Meanwhile, LUAD patients with high expression of PRKCB combined with low expression of macrophages had a significant survival advantage (Figure 10(b)) (*p* <0.05).

### PRKCB was less expressed in LUAD tumor tissues

**FIGURE 11:**
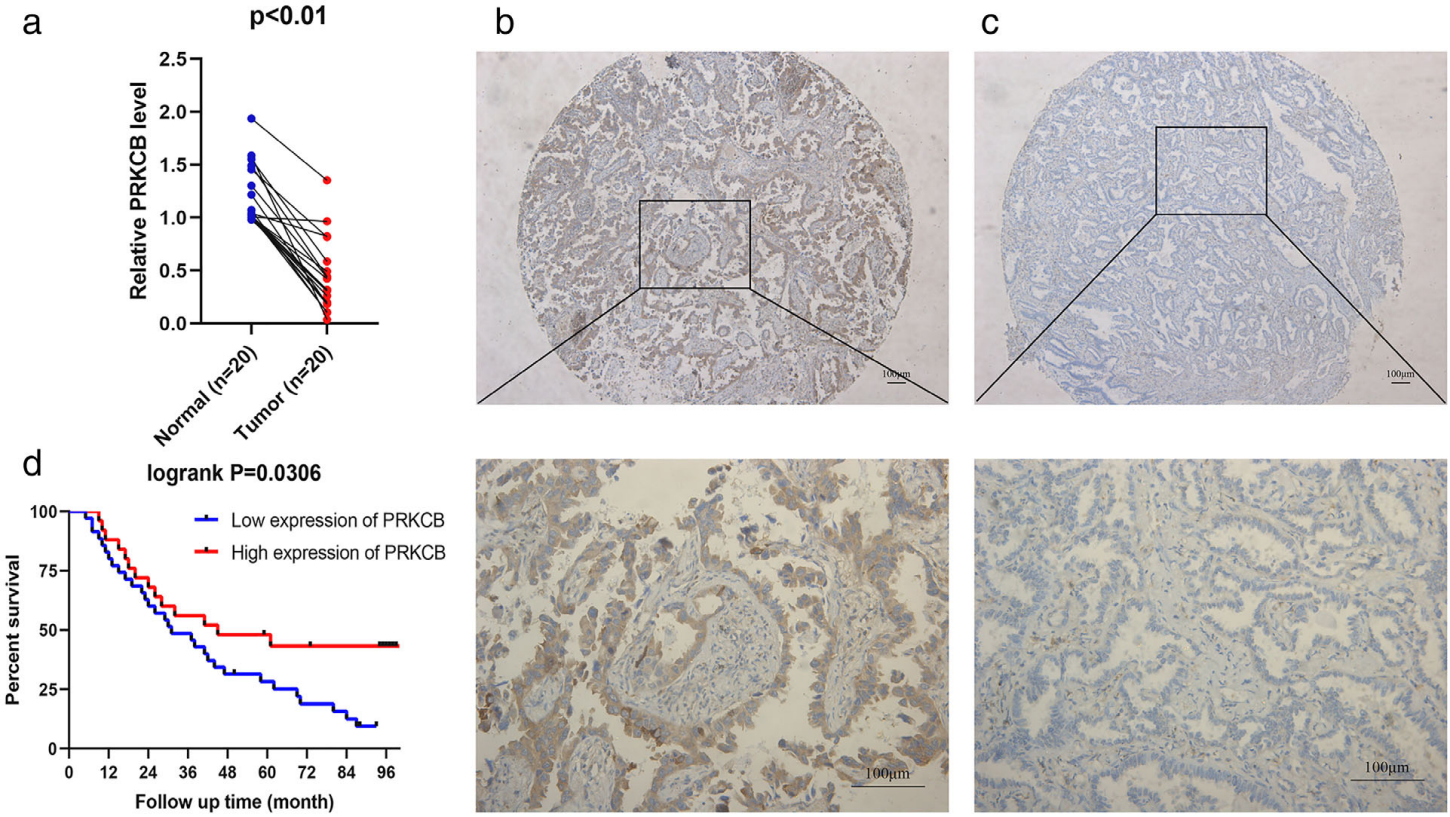
Expression of PRKCB and its clinical significance in lung adenocarcinoma (LUAD). (a) The expression of PRKCB was detected by qRT‐PCR in LUAD tissues (tumor) and adjacent normal lung tissues (normal). (b) Representative images of PRKCB‐positive IHC were captured at 50× (top) and 200× (bottom). (c) Representative images of PRKCB‐negative IHC were captured at 50× (top) and 200× (bottom). (d) Overall survival (OS) of LUAD patients according to PRKCB expression level from the tissue microarray (TMA).

We used qRT‐PCR to detect the messenger RNA (mRNA) expression of PRKCB in 20 pairs of LUAD tumors and adjacent normal lung tissues to confirm the role of PRKCB in LUAD. As shown in Figure 11(a), the expression of PRKCB in tumor tissues was significantly lower than that in normal lung tissues (*p* < 0.05). This result was consistent with the TCGA database (Figure 4).

### In LUAD patients, low PRKCB expression was associated with poor clinical prognoses

We conducted an immunohistochemical study on the tumor specimens of 60 patients with LUAD. Figure 11(b),(c), respectively showed representative images of PRKCB positive and negative staining. According to the median IHC score, the expression can be divided into high PRKCB group (25 cases) and low PRKCB group (35 cases). We next evaluated the relationship between PRKCB IHC scores and clinical characteristics in LUAD patient specimens. The expression of PRKCB had a significant correlation between age and differentiation, but had no obvious correlation between gender, smoking, or pathological TNM stage (Table 1). Survival analysis showed that patients with low expression of PRKCB in the TMA cohort had a lower overall survival rate (Figure 11(d)). This result was consistent with the TCGA database (Figure 7). Univariate and multivariate Cox analyses shown in Table 2 indicated that PRKCB gene expression level is an independent protective factor for LUAD patients.

**TABLE 1:** Relationships between PRKCB expression levels and clinicopathological characteristics of 60 patients with LUAD

	*N*	PRKCB expression		*p* value
		Low	High	
Total cases	60	35	25	
Gender				
Male	29	19	10	0.275
Female	31	16	15	
Age (y)				
<60	24	9	15	0.008
≥60	36	26	10	
Smoking				
Yes	9	7	2	0.199
No	51	28	23	
Differentiation				
Well	5	3	2	0.034
Moderate	39	27	12	
Poor	16	5	11	
Stage				
I	19	9	10	0.423
II	27	18	9	
III–IV	14	8	6	

**TABLE 2:** PRKCB expressiona associated with clinical pathological characteristics based on TMA cohort

Variables	Univariate			Multivariate		
	HR	95% CI	*p*	HR	95% CI	*p*
Age (≤65 vs. >65 y)	1.024	0.567–1.852	0.936	0.995	0.523–1.894	0.988
Gender (female vs. male)	2.321	1.271–4.239	0.006	2.669	1.278–5.573	0.009
Smoking (no vs. yes)	0.752	0.318–1.779	0.517	0.515	0.187–1.421	0.200
Stage (TNM I vs. TNM II–IV)	2.231	1.118–4.451	0.023	1.430	0.660–3.100	0.364
Differentiation (well + moderate vs. poor)	1.582	0.823–3.040	0.169	1.752	0.769–3.995	0.182
PRKCB	0.880	0.807–0.960	0.004	0.883	0.805–0.970	0.009

## DISCUSSION

With the impact on air pollution and smoking, the incidence and mortality of lung cancer continue to rise. LUAD is the most common histological subtype of NSCLC. It is often diagnosed at advanced stage because of its absence of obvious symptoms.

This year, epigenetic modification has received increasing attention, especially in cancer‐related research. DNA methylation, one of the epigenetic modifications, controls cell proliferation, differentiation, and apoptosis in eukaryotes, and directly or indirectly controls tumorigenesis.^20^ With the continuous study of gene promoter methylation, we not only have a new understanding of the mechanism of tumorigenesis, but also identified useful biomarkers through changes in gene DNA methylation, providing new methods for the diagnosis and treatment of diseases.^21^ Therefore, an in‐depth understanding of tumor suppressor genes associated with LUAD will be of great value of early clinical diagnosis and treatment of this disease.

In this study, we freely obtained a LUAD gene expression profile (GSE118370) and a LUAD methylation expression profile (GSE139032) from GEO. R software packages were used to analyze LUAD tissue and normal samples. This study was aimed to identify potential biomarkers related to aberrant methylation of LUAD to contribute to the early diagnosis and treatment of LUAD patients. In these two data, 124 overlapping genes were found, that is, 124 aberrantly methylated genes. To further analyze overlapping genes, we used R package Cluster Profiler for functional and pathway analysis. The results of GO analysis indicate that these genes have something to do with the regulation of calcium ion homeostasis and transcription activator activity. Meanwhile, KEGG pathway analysis indicated that overlapping genes are mainly concentrated in calcium signaling pathway. We constructed a PPI network with 123 nodes and 97 edges, and selected the first 10 genes as the central genes using CytoHubba, including RYR2, ADCY4, ADCY5, DRD5, PRKCB, NMUR2, ADRA2C, DRD4, SOX17, and FGF2. We used the TCGA database for further verification. As a result, ADCY4, ADCY5, FGF2, PRKCB, and SOX17 were obtained. They are all hypermethylated and underexpressed in LUAD. Next, we used TCGA database analysis to show that PRKCB and FGF2 are associated with the clinical prognosis of LUAD and are independent prognostic factors. Moreover, the effect of PRKCB and FGF2 on the OS rate of LUAD was analyzed by GEPIA. We can conclude that the low expression of PRKCB in LUAD patients has a poor prognosis. Next, we further studied the GSEA pathway of PRKCB in LUAD through the TCGA database. The results show that PRKCB is indeed involved in cancer‐related pathways and immune cell receptor signaling pathways. TIMER verified that PRKCB is associated with immune cell infiltration in LUAD. In addition, different PRKCB gene expression levels combined with different immune cell contents have an impact on the prognosis of LUAD patients. We speculate that the expression of PRKCB may affect OS by regulating the degree of immune cell infiltration in LUAD. This suggests that PRKCB may play a role in LUAD through methylation and immune cell infiltration.

PRKCB is a member of the protein kinase C (PRKC) family, which is composed of several serine/threonine kinases and can be activated by calcium and a second messenger diacylglycerol.^22^ It can be concluded from previous studies that PRKCB plays multiple roles in cell life and survival, especially in regulating cell survival and apoptosis.^23^ Studies have found that PRKCB promoters are hypermethylated in a variety of adenocarcinomas.^24^ At the same time, research indicates that PRKCB may regulate its expression in NSCLC through the Wnt signaling pathway.^25^

In summary, based on comprehensive data processing and analysis, we found that PRKCB is highly methylated and lowly expressed in LUAD and is associated with immune cell infiltration. At the same time, the survival prognosis of LUAD patients with high PRKCB expression is better. Subsequent experiments such as qRT‐PCR and IHC have also preliminarily verified this conclusion. Therefore, PRKCB is relevant to prognosis of LUAD through methylation and immune infiltration. Moreover, because gene methylation modifications usually occur in the early stage of cancers, the methylation change of the PRKCB gene may occur in the early stage of LUAD, which may have certain value for the early diagnosis of LUAD patients.^26^ Of course, the current research is still limited, and further research is needed. The expression of PRKCB is accompanied by a large number of immune cell infiltrations, which means that PRKCB may play an important role in the tumor microenvironment of LUAD by regulating the tumor infiltration of immune cells.^27^ This provides new ideas for future antitumor immunotherapy and anti‐drug resistance in LUAD.

## CONFLICT OF INTEREST

The authors declare that they have no conflicts of interests.

## Data Availability

The datasets generated during and/or analyzed during the current study are available from GEO (https://www.ncbi.nlm.nih.gov/geo/) and TCGA official website (https://portal.gdc.cancer.gov/repository).
